# Perceived factors and barriers affecting physiotherapists’ decision to use spinal manipulation and mobilisation among infants, children, and adolescents: an international survey

**DOI:** 10.1080/10669817.2024.2363033

**Published:** 2024-06-28

**Authors:** Jenifer L. Dice, Jean-Michel Brismee, Frédéric P. Froment, Janis Henricksen, Rebecca Sherwin, Jan Pool, Nikki Milne, Derek Clewley, Annalie Basson, Kenneth A. Olson, Anita R. Gross

**Affiliations:** a School of Physical Therapy, Texas Woman’s University, Houston, TX, USA; b Center for Rehabilitation Research, School of Health Professions, Texas Tech University Health Sciences Center, Lubbock, TX, USA; c International Academy of Musculoskeletal Physiotherapy, Paris, France; d School of Physical Therapy, University of St. Augustine, Dallas, TX, USA; e NELA Rehabilitation, Physical Therapy Department, West Monroe, Louisiana, USA; f Hauser School of Physical Therapy, University of the Cumberlands, Williamsburg, KY, USA; g Institute of Movement Studies, University of Applied Sciences, Utrecht, The Netherlands; h THINK Paediatrics Research Group, Faculty of Health Sciences and Medicine, Bond University, Robina, Queensland, Australia; i International Organisation of Physiotherapists in Paediatrics (IOPTP); j Doctor of Physical Therapy Division, Duke University, Durham, North Carolina, USA; k Faculty of Health Sciences, Physiotherapy Department, University of the Witwatersrand, Johannesburg, South Africa; l Northern Rehab Physical Therapy Specialists, DeKalb, IL, USA; m Rehabilitation Sciences, McMaster University, Hamilton, Canada

**Keywords:** Paediatrics, physiotherapy, physical therapy, cervical, thoracic, lumbar

## Abstract

**Objective:**

To identify factors and barriers, which affect the utilisation of spinal manipulation and mobilisation among infants, children, and adolescents.

**Methods:**

Twenty-six international expert physiotherapists in manual therapy and paediatrics were invited to participate in a Delphi investigation using Qualtrics^Ⓡ^. In Round-1 physiotherapists selected from a list of factors and barriers affecting their decision to use spinal manipulation and mobilisation in the paediatric population and had opportunity to add to the list. Round-2 asked respondents to select as many factors and barriers that they agreed with, resulting in a frequency count. The subset of responses to questions around barriers and facilitators are the focus of this study.

**Results:**

Twelve physiotherapists completed both rounds of the survey. Medical diagnosis, mechanism of injury, patient presentation, tolerance to handling, and therapist’s knowledge of techniques were the dominant deciding factors to use spinal manipulation and mobilisation among infants, children, and adolescents across spinal levels. More than 90% of the respondents selected manipulation as inappropriate among infants as their top barrier. Additional dominant barriers to using spinal manipulation among infants and children identified by ≥ 75% of the respondents included fear of injuring the patient, fear of litigation, lack of communication, lack of evidence, lack of guardian consent, and precision of the examination to inform clinical reasoning.

**Conclusion:**

This international survey provides much needed insight regarding the factors and barriers physiotherapists should consider when contemplating the utilisation of spinal mobilisation and manipulation in the paediatric population.

## Introduction

Orthopaedic manipulative physiotherapy (OMPT) incorporates a range of therapeutic approaches, including manipulation and mobilisation techniques. Mobilisation is defined by the International Federation of Orthopaedic Manipulative Physical Therapists (IFOMPT) as ‘a continuum of skilled passive movements applied at varying speeds and amplitudes to joints, muscles or nerves with the intent to restore optimal motion, function and reduce pain.’ [1] Additionally, IFOMPT defines manipulation as ‘a passive, high velocity, low amplitude (HVLA) thrust applied to a joint complex within its anatomical limit with the intent to restore optimal motion, function and to reduce pain.’ [1] Internationally, varied theoretical views regarding utilisation of spinal manipulation and mobilisation among infants (birth to < 2-years), children (2 to 12-years), and adolescents (13 to < 18-years) have been proposed [2]. Evidence regarding the use of these techniques in paediatric populations is limited, especially regarding physiotherapy techniques, as opposed to other professional techniques (e.g. chiropractic, osteopathic, etc.) and there are safety concerns [3]. Outlining the current scientific evidence and acquiring input from clinical experts regarding the use of spinal manipulation and mobilisation among infants, children, and adolescents to inform evidence-based practice is imperative. Identifying the factors and barriers that influence clinical experts’ clinical reasoning and treatment planning is an important step toward deeper understanding of the rationale used by physiotherapists when incorporating spinal manipulation and mobilisation into their treatment of infants, children, and adolescents.

Adverse events from spinal manipulation or mobilisation with paediatric populations are inadequately reported in the scientific literature; however, mild to severe adverse events (including life-threatening conditions and death) have been documented [3]. Systematic reviews undertaken by Driehuis et al. [2] and Milne et al. [3] have unveiled significant adverse events including rib fractures, acute respiratory decompensation, neurological deficits, and subarachnoid hemorrhage resulting in fatality. The practice of OMPT for infants, children, and adolescents internationally is based on existing guidelines, protocols, and policy statements; albeit lacking high-quality evidence to support its clinical use [3]. Gaining an understanding of what barriers and factors expert paediatric and manual physiotherapists consider when treating vulnerable paediatric populations is an important part of clinical reasoning, which could help other clinicians.

An international Delphi investigation was designed to build consensus regarding the use of spinal mobilisation and manipulation among infants, children, and adolescents with varying conditions and impairments [4]. Included within the Delphi investigation, the researchers sought additional insight regarding the factors and barriers identified by the expert physiotherapists, which influence their decision to use or not use spinal manipulation and mobilisation in paediatric populations. Although such factors and barriers have been reported by expert physiotherapists in the United States regarding the use of manual therapy [5], they have not been reported on an international level specific to spinal mobilisation and manipulation in paediatric populations. Expert physiotherapists in the United States identified the following factors when considering the use of mobilisation or manipulation: concerns regarding soft tissue and/or skeletal integrity; diagnosis of the patient; imaging needed prior to treatment; patient presentation; tolerance to handling; mechanism of injury; and therapist’s knowledge of spinal mobilisation/manipulation and/or when to use the technique [5]. Barriers identified included: fear of injuring patient; fear of litigation; lack of knowledge or training on technique; lack of evidence to support the use of spinal mobilisation/manipulation; lack of experience in paediatric development; and bias against using spinal mobilisation/manipulation [5]. Therefore, the purpose of this survey was to report the perceived factors and barriers to performing spinal mobilisation and manipulation among a panel of international expert physiotherapists.

## Methods

### Study design & recruitment

The study design of the larger Delphi investigation has been previously reported [4]. A set of questions were developed to investigate the factors and barriers identified when considering the use of spinal manipulation and mobilisation in infants, children, and adolescents. These survey questions were a subset of questions from Rounds 1 and 2 of the previously reported larger Delphi investigation [4]. The data gathered from these questions did not follow the formal Delphi procedure and are therefore being reported separately in this manuscript. Prior to participant recruitment, Institutional Review Board (IRB) exemption was awarded by the Texas Tech University Health Sciences Center (TTUHSC), USA (#L21–151), and ethical approval was obtained by the Bond University Human Research Ethics Committee, Australia (#NM03322).

### Survey participants

Twenty-six expert physiotherapists from Australia, Belgium, Canada, Republic of Korea, South Africa, the Netherlands, United Kingdom, and the United States were identified and invited to participate via e-mail. Invited expert physiotherapists were selected based on academic and clinical experience and expertise [4]. For the purposes of the primary Delphi investigation, an ‘expert’ was delineated as a Physiotherapist proficient in the English language who either specialised in the exclusive treatment of paediatric orthopaedic conditions utilising manual or manipulative therapy as a component of their treatment approach, possessed specialised education in musculoskeletal manipulative therapy with a minimum of five years of clinical practice, focused on paediatrics with exposure to manual therapy techniques and a minimum of five years of clinical practice, or held a research background, inclusive of publications in the field of paediatrics and manipulative therapy. They voluntarily participated in each round. Survey responses remained anonymous and incomplete surveys were excluded from data analysis.

### Survey questions Round-1

**Figure 1.**
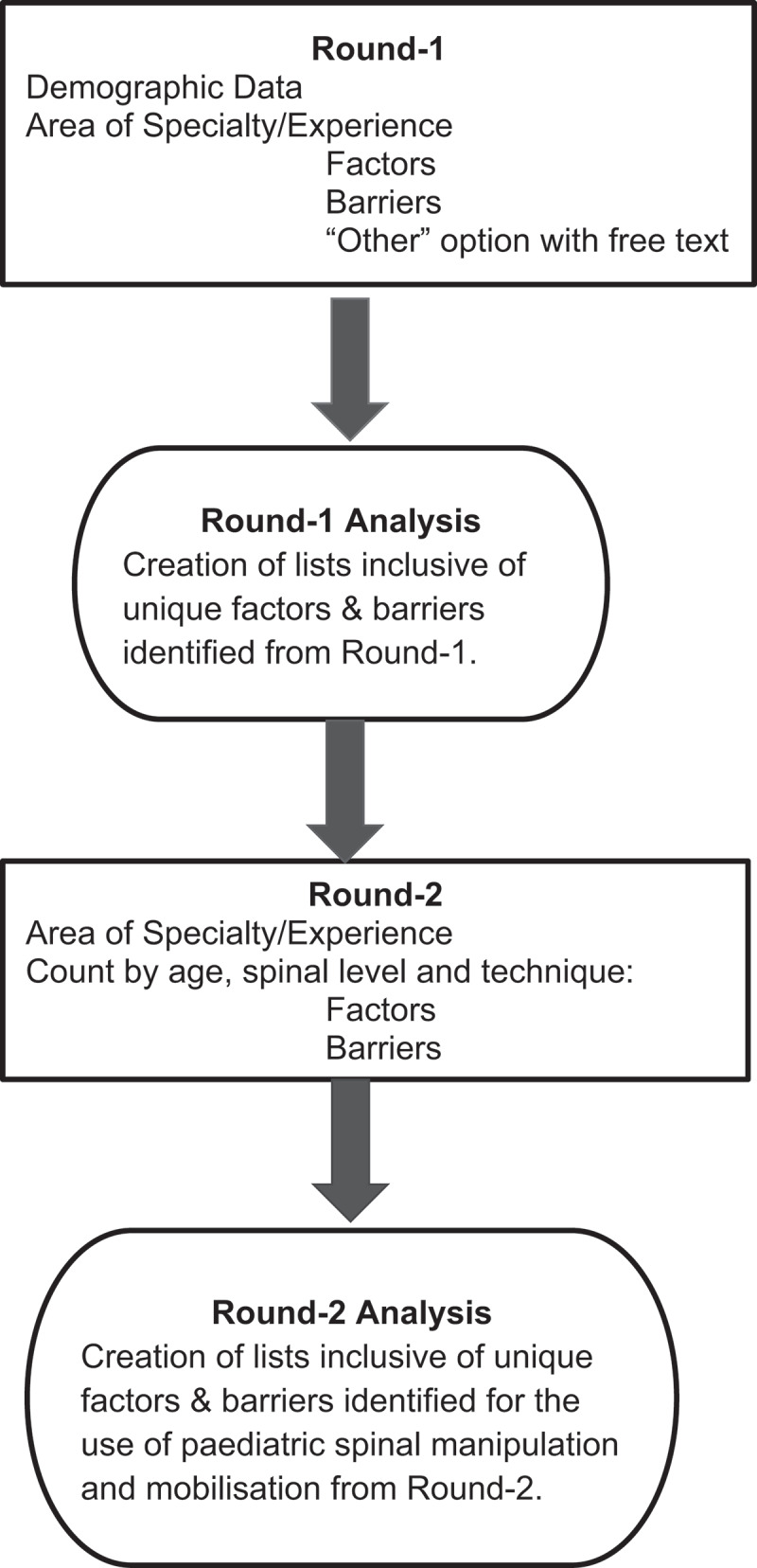
Process for identifying factors and barriers.

Round-1 included limited demographic data (i.e. years of experience, country of origin, education, and teaching experience), area of specialisation (adults (≥18 years), paediatrics (<18 years) or both (adults and paediatrics), and a list of factors and barriers identified by United States expert physiotherapists [5]. The panel had the opportunity to provide additional factors and barriers as they felt necessary (Figure 1).

### Identification of Round-1 variables

Two investigators (JD and RS) reviewed the additional factors and barriers identified by the Round-1 respondents to determine the complete list of factors and barriers presented in Round-2 (Figure 1). If there was disagreement for any item, a third investigator (JH) evaluated the data independently. A majority vote (2 out of 3 investigators) determined the final factors and barriers lists presented in Round-2 (Table 1).

**Table 1.** Round-2 factors and barriers presented to physiotherapists regarding the use of spinal mobilisation and manipulation for the paediatric population.

Factors	Barriers
Concerns regarding soft tissue and/or skeletal integrity	It is inappropriate to use mobilisations/manipulations for this age.
Medical diagnosis of patient	There are no preventative barriers to mobilisations/manipulations
Imaging needed prior to spinal mobilisations/manipulations	Fear of injuring the patient (e.g. causing neurovascular or bony injury)
Informed consent from a guardian	Fear of litigation
Informed assent from the patient	Lack of communication (among patient, guardian, and therapist)
Mechanism of injury	Lack of evidence to support the use of mobilisation/manipulation in the age group
Patient presentation (e.g. agitation, engagement, state of arousal)	Lack of guardian consent
Patient’s tolerance to handling	Lack of patient assent
Posture exercises and self- mobilisations/manipulations are not enough to treat effectively	Lack of experience in paediatric development
Therapist’s knowledge of techniques and when to use them appropriately	Lack of knowledge/training in techniques
	Lack of mentorship
	Medical diagnosis is not clearly defined
	Poor understanding/cognition on the part of patient or guardian
	The precision of examination to inform clinical reasoning
	Mobilisation/manipulation is not within the scope of practice

### Survey questions Round-2

Round-2 was sent to the e-mail addresses provided by the respondents at the end of Round-1 (*n* = 21). The expert physiotherapists were asked their area of specialisation. Participants were then asked to select the factors and barriers they perceived to affect their decision to use or not use spinal manipulation and mobilisation by spinal level (cervical, thoracic, and lumbar) and age (<2, 2–12, <18 years). There was no minimum or maximum set; respondents could choose as few, or as many as they deemed appropriate (Table 1).

### Data Analysis

Microsoft Excel (version 365) was used to collate and analyse the data. Frequency counts were calculated for the demographic variables, which included the survey respondents’ physiotherapy specialisation. Additionally, the frequency of responses by techniques (mobilisation and manipulation), age, and spinal level were calculated for Round-2.

## Results

### Demographics of respondents

Of the twenty-six experts invited, twenty-one completed the Round-1 survey (Table 2). One respondent was excluded from analysis because of his/her osteopathic medicine degree as the main objective of this survey was to quantify physiotherapy practice. Twelve respondents completed the survey in Round-2, resulting in a 40% attrition rate. Of the twelve, three reported treating mainly adults, five reported treating mainly patients from birth up to 18 years, and four reported treating patients above and below the age of 18 years. For the purpose of clarity, dominant factors and barriers were defined as ≥ 75% respondents selecting that item.

**Table 2.** Demographics of respondents.

Characteristic	Count (%) Round-1 *n* = 20
Female	14 (70)
Male	6 (30)
Age in years, −X [SD]	49.6 [11.2]
Years of practice −X [SD]	23.3 [12.1]
Number of Countries represented	8
Australia	3
Belgium	1
Canada	2
Korea	2
Netherlands	3
South Africa	1
United Kingdom	2
United States of America	6
**Specialisation**	
Adult	2
Both	10
Paeds	8
**Highest Physiotherapy Education Level (%)**	
Bachelor of Science in Physiotherapy	1 (5)
Master of Science in Physiotherapy	5 (25)
Clinical Doctorate of PT/Masters (extended)	3 (15)
Post-Professional Doctorate Degree in PT	11 (55)
Advanced Certification in Orthopaedic Manual Therapy (OMT)	8 (40)
Advanced Certification in Paediatrics	6 (30)
Advanced Certification in OMT & Paediatrics	3 (15)
**Teaching Setting (%)**	
Not currently teaching physiotherapy	3
Academic	12
Continuing Education	7
Residency/Fellowship	1
Clinical Setting (clinical instructor)	7

PT: Physiotherapy, OMT: Orthopaedic Manipulative Therapy, SD: Standard Deviation.

### Factors affecting physiotherapists’ decision to use or not use spinal mobilisation or manipulation

When considering the use of spinal mobilisation and manipulation, the physiotherapists reported that the patient presentation (>90%), tolerance to handling (>90%), mechanism of injury (>80%), medical diagnosis of the patient (≥75%), and therapist’s knowledge of techniques (≥75%) were the dominant deciding factors to use these techniques across all paediatric ages and spinal levels (Table 3). The respondents identified concerns regarding soft tissue and skeletal integrity as a dominant factor across all spinal levels for infants (>90%) and children (>80%) when considering the use of spinal mobilisation and manipulation and among adolescents (≥75%) when considering spinal manipulation. Guardian consent (>90%) was identified as a dominant factor across all spinal levels for infants and children when considering the use of spinal manipulation only, whereas informed patient assent (≥75%) was also identified when considering manipulation for children and adolescents across spinal levels.

**Table 3.** Factors affecting physiotherapists’ decision to use or not use spinal mobilisation and manipulation for the paediatric population.

	Mobilisation									Manipulation								
Age (years)	<2			2–12			13–18			<2			2–12			13–18		
Region	C	T	L	C	T	L	C	T	L	C	T	L	C	T	L	C	T	L
Concerns regarding soft tissue and/or skeletal integrity	**✔**	**✔**	**✔**	**✔**	**✔**	**✔**	#	#	#	**✔**	**✔**	**✔**	**✔**	**✔**	**✔**	**✔**	**✔**	**✔**
Medical diagnosis of patient	**✔**	**✔**	**✔**	**✔**	**✔**	**✔**	#	**✔**	**✔**	**✔**	**✔**	**✔**	**✔**	**✔**	**✔**	**✔**	**✔**	**✔**
Imaging needed prior to spinal mobilisations/manipulations	^	^	^	^	^	^	^	^	^	#	#	#	#	#	#	^	^	^
Informed consent from a guardian	#	#	#	**✔**	**✔**	**✔**	#	#	#	**✔**	**✔**	**✔**	**✔**	**✔**	**✔**	**✔**	#	#
Informed assent from the patient	^	^	^	#	#	#	**✔**	**✔**	**✔**	#	#	#	**✔**	**✔**	**✔**	**✔**	**✔**	**✔**
Mechanism of injury	**✔**	**✔**	**✔**	**✔**	**✔**	**✔**	**✔**	**✔**	**✔**	**✔**	**✔**	**✔**	**✔**	**✔**	**✔**	**✔**	**✔**	**✔**
Patient presentation (e.g., agitation, engagement, state of arousal)	**☆**	**✔**	**✔**	☆	☆	☆	☆	☆	☆	☆	☆	☆	☆	☆	☆	**✔**	**✔**	**✔**
Patient’s tolerance to handling	☆	☆	☆	☆	☆	☆	**✔**	**✔**	**✔**	☆	☆	☆	☆	☆	☆	**✔**	**✔**	**✔**
Posture exercises and self-mobilisations/manipulations are not enough to treat effectively	^	^	^	#	#	#	#	#	#	^	^	^	^	^	^	#	#	#
Therapist’s knowledge of techniques and when to use them appropriately	**✔**	**✔**	**✔**	**✔**	**✔**	**✔**	**✔**	**✔**	**✔**	**✔**	**✔**	**✔**	**✔**	**✔**	**✔**	**✔**	**✔**	**✔**

C = cervical; T = thoracic; L = lumbar; selected by 100% (☆); selected by with ≥ 75% (**✔**); between 50 and 74% (#); between 25 and 49% (^); between 1 and 24% (*); and 0% (blank, no symbol) of respondents.

### Barriers preventing physiotherapists’ from using spinal mobilisation or manipulation

The dominant barriers preventing the use of spinal mobilisation and manipulation across spinal levels among infants and children were identified by the survey respondents as lack of knowledge or training in techniques (infants 100%, children > 80%), lack of mentorship (>80%), the medical diagnosis not being clearly defined (≥75% mobilisation, >90% manipulation), and poor understanding/cognition on the part of the patient or guardian (≥75%). All respondents (100%) indicated cervical manipulation was inappropriate in infants (<2 years). Likewise, > 90% of the respondents indicated that thoracic and lumbar manipulation was inappropriate to treat infants. Additionally, with regards to spinal manipulation, the dominant barriers identified among infants and children were fear of litigation (>90%), fear of injuring the patient (≥75%), lack of communication (≥75%), lack of evidence (infants >90%, ≥75% children), lack of guardian consent (≥75%), and precision of the examination to inform clinical reasoning (≥75%) (Table 4). The vast majority of respondents identified lack of knowledge or training in techniques (>80%) and medical diagnosis not being clearly defined (>90%) when considering manipulation for adolescents as dominant barriers.

**Table 4.** Barriers preventing physiotherapists from using spinal mobilisation and manipulation in the paediatric population.

	Mobilisation									Manipulation								
Age (years)	<2			2–12			13–18			<2			2–12			13–18		
Region	C	T	L	C	T	L	C	T	L	C	T	L	C	T	L	C	T	L
It is inappropriate to use mobilisations/manipulations for this age	#	#	#	^	^	^	*	*	*	☆	**✔**	**✔**	#	#	#	^	^	^
There are no preventative barriers to mobilisations/manipulations	*	*	*	*	*	*	^	^	^	^	^	^	^	^	^	*	*	*
Bias against using mobilisations/manipulations	#	#	#	*	*	*	*	*	*	**✔**	**✔**	#	**✔**	**✔**	#	#	#	^
Fear of injuring patient (e.g. causing neurovascular/bony injury)	**✔**	**✔**	**✔**	#	#	#	#	^	^	**✔**	**✔**	**✔**	**✔**	**✔**	**✔**	#	#	#
Fear of litigation	#	**✔**	**✔**	#	#	#	#	^	^	**✔**	**✔**	**✔**	**✔**	**✔**	**✔**	#	#	#
Lack of communication (among patient, guardian, and therapist)	#	#	#	#	#	#	^	^	^	**✔**	**✔**	**✔**	**✔**	**✔**	**✔**	#	#	^
Lack of evidence to support mobilisations/manipulations use	#	#	#	#	#	#	^	^	^	**✔**	**✔**	**✔**	**✔**	**✔**	**✔**	#	#	#
Lack of guardian consent	#	#	#	#	#	#	#	#	#	**✔**	**✔**	**✔**	**✔**	**✔**	**✔**	#	#	#
Lack of patient assent	#	#	#	#	#	#	#	#	#	**#**	**#**	**#**	**#**	**✔**	**✔**	#	#	#
Lack of experience in paediatric development	**✔**	**✔**	**✔**	#	#	#	#	#	#	**✔**	**✔**	**✔**	**✔**	**✔**	**✔**	**✔**	#	#
Lack of knowledge/training in techniques	☆	☆	☆	**✔**	**✔**	**✔**	#	#	#	**✔**	**✔**	**✔**	**✔**	**✔**	**✔**	**✔**	**✔**	**✔**
Lack of mentorship	**✔**	**✔**	**✔**	**✔**	**✔**	**✔**	#	#	#	**✔**	**✔**	**✔**	**✔**	**✔**	**✔**	**✔**	**✔**	#
Medical diagnosis is not clearly defined	**✔**	**✔**	**✔**	**✔**	**✔**	**✔**	#	#	#	**✔**	**✔**	**✔**	**✔**	**✔**	**✔**	**✔**	**✔**	**✔**
Poor understanding/cognition on the part of patient or guardian	**✔**	**✔**	**✔**	**✔**	**✔**	**✔**	#	#	#	#	#	#	**✔**	**✔**	**✔**	#	#	#
Mobilisation/manipulation is not within the scope of practice	^	^	^	*	*	*				#	#	#	^	^	^	^	*	*
The precision of examination to inform clinical reasoning	#	#	#	#	#	#	#	#	#	**✔**	**✔**	**✔**	**✔**	**✔**	**✔**	#	#	#

C = cervical; T = thoracic; L = lumbar; selected by 100% (☆); 75 to 99% (✔); between 50 and 74% (#); between 25 and 49% (^); between 1 and 24% (*); and 0% (blank, no symbol) of respondents.

## Discussion

This international Delphi investigation established the opinions of expert physiotherapists regarding the factors and barriers affecting their decision to use spinal mobilisation and manipulation in paediatric populations. The international perspective may demonstrate variations in education, practice, customs and legislation between countries [6]. These countries have different health systems as compared to the United States. The dominant factors identified regarding the use of spinal mobilisation and manipulation in paediatrics included medical diagnosis of the patient, mechanism of injury, patient presentation, knowledge of technique, and patient’s tolerance to handling. There were more dominant barriers identified when considering the use of spinal manipulation among infants and children as opposed to mobilisation and the adolescent population.

### Agreement and disagreement with other studies

Overall, our findings align with a Delphi investigation of the United States physiotherapists’ utilisation of manual therapy techniques among pre-adolescent children [5]. However, unlike the 2021 US Delphi investigation [5] the current study further classified ‘pre-adolescent’ into ‘infant’ and ‘children.’ The clinical judgment of this expert panel is supported by a systematic scoping review [3]. This review reported the potential for severe harm to infants during high velocity low amplitude manipulation [3]. The respondents, in the concurrent Delphi investigation did not support the use of spinal manipulation for infants [4]. Additionally, this study looked at factors and barriers considered when treating adolescents, not previously investigated [5].

### Overall completeness and applicability of the survey findings

The generalisability of some of our results was limited. The expansion of ‘pre-adolescent’ groups (including children and infants, which were considered one single group in the previous American survey [5] gave current survey respondents the opportunity to identify the item *spinal manipulation is inappropriate* as the most significant barrier among infants. Furthermore, the opinions were more diverse regarding the perceived barriers for adolescents compared to infants and children. For example, 90 to 100% of the respondents felt spinal manipulation was inappropriate for all spinal levels in infants, compared to only 33% of the respondents answering the same for adolescents. Because opinions were diverse, it is important to not generalise the perceived barriers for the use of spinal mobilisation and manipulation in infants and children to the adolescent population.

Interestingly, 58% of expert physiotherapists in the current study reported spinal manipulation performed on infants was not within their scope of practice. This likely reflects practice acts and regulatory issues as spinal manipulation for children under the age of 2 years in countries/states/jurisdictions such as Australia was prohibited for chiropractors at the time of this survey [6], while in a small percentage of countries/states/jurisdictions, physiotherapists are not allowed to perform manipulation [7]. This also could be a result of expert physiotherapists’ self-regulation due to a lack of training and expertise in spinal manipulation use for infants. However, lack of communication and guardian consent were reported as dominant barriers affecting the use of spinal manipulation in infants and children. Such findings are similar to those reported in the chiropractic literature where issues related to patient communication and consent require adaptation and special skills from the clinicians before treatment is undertaken [8,9].

This international survey illustrates that while some factors and barriers were identified by ≥ 75% of the respondents, some were not, indicating various considerations based on the unique combination of the treating physiotherapist and patient’s needs and presentation. Since most respondents indicated that spinal mobilisation and manipulation (58% and > 90%, respectively) were inappropriate for infants, additional research should focus on children >2 years of age. Future research might also consider further subcategorisation of ‘infants’ and ‘children’ into ‘infants (<3 years),’ ‘young children (3 up to 6 years)’ and ‘older children (6 to 12 years)’, since factors and barriers (such as communication, understanding, and patient assent) may be unique based on a child’s overall cognitive, language, social-emotional, and self-help developmental milestones, all of which would need to be appropriately assessed using valid measures of development, for example the Hawaii Early Learning Profile, or other similar tools [10].

Advanced education and training could help minimise perceived factors and barriers such as the lack of knowledge of spinal mobilisation and manipulation techniques. In addition to learning the techniques, understanding how and when to apply them within the context of paediatric development is an additional layer to consider. Lastly, the lack of mentorship could apply to ‘paediatric’ physiotherapists learning spinal mobilisation and manipulation techniques. Physiotherapists treating an adult population or both adult and paediatric populations may need a deeper understanding of infant and child development as well as training in tools to assess their global development.

### Strengths

This study followed strict methodological guidelines for e-surveys (Checklist for Reporting Results of Internet E-Surveys (CHERRIES) [11]. The present study was embedded within a larger Delphi investigation and as such the expert international physiotherapists who answered Round-2 were participants in Round-1 demonstrating consistency of expert opinion. This study categorised and sought information on three distinct paediatric age groups. The heterogeneity of the sample including physiotherapists who treat adult and paediatric clients is a strength. The expert physiotherapists worked with diverse caseloads and thus offered unique perspectives.

### Limitations

As this study of factors and barriers was embedded within a larger Delphi survey, the overall attrition rate of 40% between Round-1 and Round-2 was likely a direct result of the large number of items included in the survey [12]. In Round-1 we asked approximately 40 questions and Round-2 included a minimum of 396 items that required scoring. It is possible that respondents dropped out due to the sheer volume of questions, which included both the Delphi and the embedded factors and barriers survey. Because this study did not follow the Delphi process but included 2 rounds, information reflected back to the participants from Round-1 to Round-2 could have biased some of their answers. Furthermore, because the initial number of experts were required to have mastery of the English language, this inherently limited participants and could limit the external validity of the findings.

## Conclusion

This international survey provides much needed insight regarding the factors and barriers physiotherapists should consider when contemplating the utilisation of spinal mobilisation and manipulation in paediatric populations.

*Implications for practice*: It is possible that some perceived barriers may be addressed by advanced education and training.

*Implications for research*: Additional research among children should consider further sub-categorisation of ages based on cognitive, language, social-emotional, and self-care developmental milestones.

## Supplementary Material

Appendix B Survey_Round_2 Clean.docx

Appendix A Survey_Round_1 Clean.docx

## References

[1] Rushton A, Beeton K, Jordaan R, et al. Standards Document. Published online 2016. [cited 2024 Jan 12]. Available from: https://www.ifompt.org/site/ifompt/IFOMPT%20Standards%20Document%20definitive%202016.pdf

[2] Driehuis F, Hoogeboom TJ, Nijhuis-van der Sanden MWG, et al. Spinal manual therapy in infants, children and adolescents: A systematic review and meta-analysis on treatment indication, technique and outcomes. PLoS One. 2019;14(6):e0218940. doi: 10.1371/journal.pone.0218940 31237917 PMC6592551

[3] Milne N, Longeri L, Patel A, et al. Spinal manipulation and mobilisation in the treatment of infants, children, and adolescents: a systematic scoping review. BMC Pediatr. 2022;22(1):721. doi: 10.1186/s12887-022-03781-6 36536328 PMC9762100

[4] Brismée JM, Froment FP, Henricksen L, et al. Spinal manipulation and mobilisation among infants, children, and adolescents: an international Delphi survey of expert physiotherapists. J Manual Manipulative Ther. 2024;1–11. doi: 10.1080/10669817.2024.2327782 PMC11216234 38484120

[5] Dice JL, Dendy D, Sizer PS, et al. Manual therapy in preadolescent children: a delphi investigation of physical therapists in the United States. Phys Ther. 2021;101(4):zab027. doi: 10.1093/ptj/pzab027 33513233

[6] Safer Care Victoria. Chiropractic spinal manipulation of children under 12- Independent review Melbourne 2019. Available from: https://www.safercare.vic.gov.au/sites/default/files/2019-10/20191024-Final%20Chiropractic%20Spinal%20Manipulation.pdf

[7] Froment FP, Olson KA, Hooper TL, et al. Large variability found in musculoskeletal physiotherapy scope of practice throughout WCPT and IFOMPT affiliated countries: An international survey. Musculoskelet Sci Pract. 2019 Jul;42:104–119. doi: 10.1016/j.msksp.2019.04.012 31102821

[8] Hawk C, Schneider MJ, Vallone S, et al. Best practices for chiropractic care of children: a consensus update. J Manipulative Physiol Ther. 2016;39(3):158–168. doi: 10.1016/j.jmpt.2016.02.015 27040034

[9] Keating G, Hawk C, Amorin-Woods L, et al. Clinical practice guideline for best practice management of pediatric patients by chiropractors: results of a delphi consensus process. J Integr Complement Med. [2023 Oct 30];30(3):216–232. doi: 10.1089/jicm.2023.0010 Epub ahead of print. PMID: 37902954. 37902954 PMC10954607

[10] VORT Corporation. Revised Hawaii early learning profile – HELP charts. 2016.

[11] Bennett C, Khangura S, Brehaut JC, et al. Reporting guidelines for survey research: an analysis of published guidance and reporting practices. PLoS Med. 2011;8(8):e1001069. doi: 10.1371/journal.pmed.1001069 PMC3149080 21829330

[12] Gargon E, Crew R, Burnside G, et al. Higher number of items associated with significantly lower response rates in COS Delphi surveys. J Clin Epidemiol. 2019;108:110–120. doi: 10.1016/j.jclinepi.2018.12.010 30557677 PMC6438267

