# Supplementary material for: Perceived factors and barriers affecting physiotherapists’ decision to use spinal manipulation and mobilisation among infants, children, and adolescents: an international survey
Source: J Man Manip Ther. 2024 Jun 28;32(3):295–303. doi: 10.1080/10669817.2024.2363033 (PMC11216267; doi:10.1080/10669817.2024.2363033)
Supplement: Appendix B Survey_Round_2 Clean.docx [file YJMT_A_2363033_SM3351.docx]

Survey Round 2

Please indicate your level of education by selecting all that apply:

- Non-university diploma in Physiotherapy


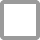
 Bachelor of Science in Physiotherapy


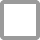
 Master of Science in Physiotherapy

- Clinical doctorate (DPT) or extended Master degree in Physiotherapy


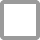
 Post-professional doctorate (e.g., PhD, EdD, ScD)

- Advanced certiﬁcation in orthopaedic manual therapy (OMT)


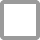
 Advanced certiﬁcation in paediatrics


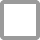
 Osteopathic Medicine degree or certiﬁcation

Please indicate your level of education by selecting all that apply:

- Non-university diploma in Physiotherapy


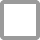
 Bachelor of Science in Physiotherapy


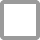
 Master of Science in Physiotherapy

- Clinical doctorate (DPT) or extended Master degree in Physiotherapy


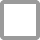
 Post-professional doctorate (e.g., PhD, EdD, ScD)

- Advanced certiﬁcation in orthopaedic manual therapy (OMT)


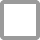
 Advanced certiﬁcation in paediatrics


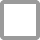
 Osteopathic Medicine degree or certiﬁcation

Please select your primary practice


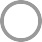
 Adult population (>18 years)


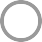
 Paediatric population (<18 years)


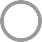
 Both (adult and paediatric populations)

For the purposes of this survey, consider the following definitions (IFOMPT Standards Document, 2016):


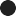
 **Mobilisation** is “a continuum of skilled passive movements that are applied at varying speeds and amplitudes to joints, muscles or nerves with the intent to restore optimal motion, function, and/or to reduce pain”.


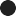
 **Manipulation** is “a passive, high velocity, low amplitude thrust applied to a joint complex within its anatomical limit with the intent to restore optimal motion, function, and/or to reduce pain”.

The following section will address the **FACTORS** and **BARRIERS** identified from Round 1 for each spinal level and age group regarding the utilisation of mobilisations and manipulation.

- Infants (birth to <2 years)
- Children (2 to 12 years)
- Adolescents (13 to <18 years)

You are not required to make a selection in every row and column.

Once satisfied with your answered item(s), please select "Continue without answering"

Please select the **FACTORS** that you believe would AFFECT a therapist’s decision to use or not use spinal **MOBILISATIONS** among the specific ages and spinal levels.


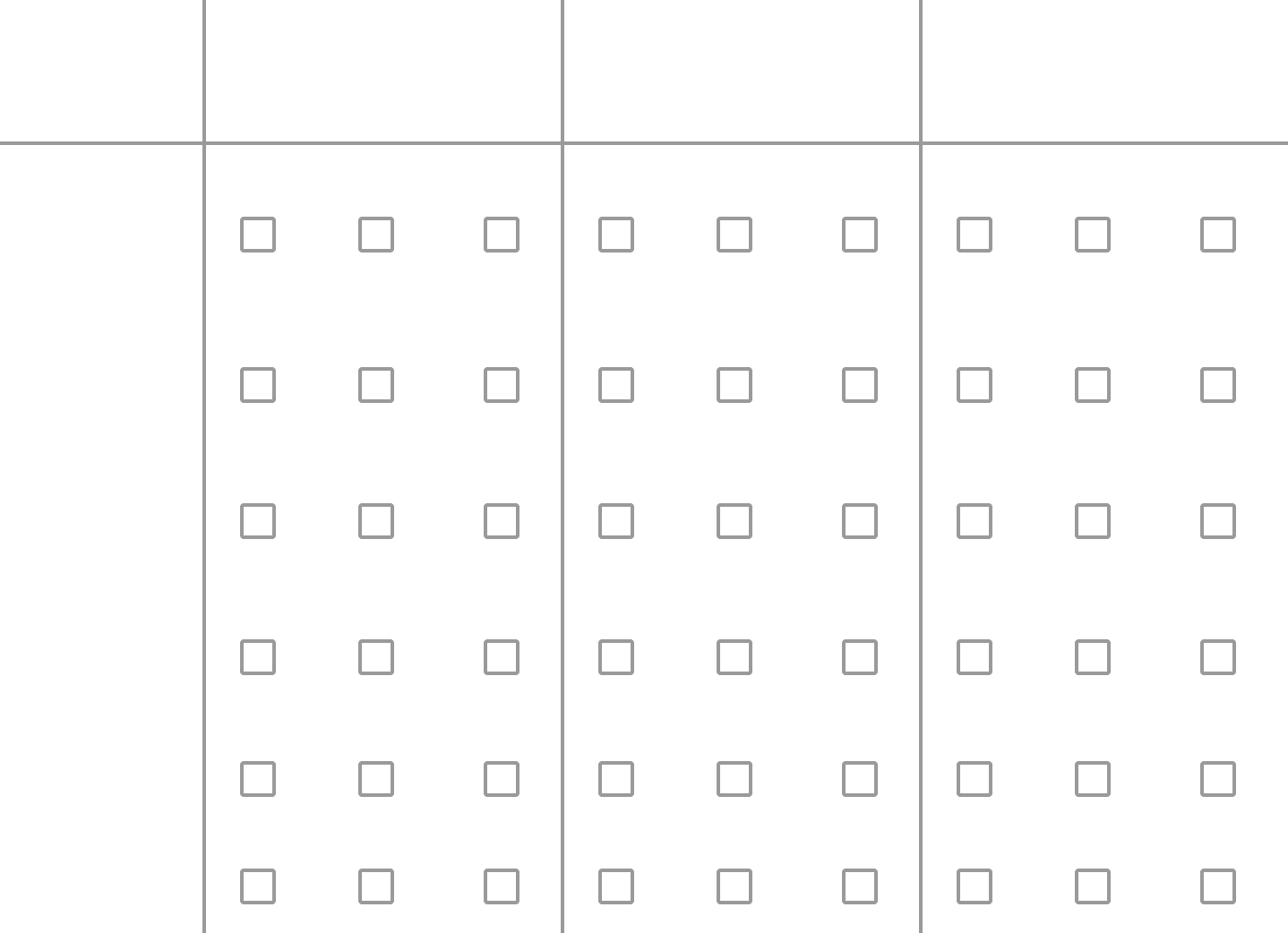


|  |  | **Cervical** |  |  | **Thoracic** |  |  | **Lumbar** |
| --- | --- | --- | --- | --- | --- | --- | --- | --- |
|  | <2 | 2-12 | <18 | <2 | 2-12 | <18 | <2 | 2-12 <18 |
| Concerns regarding soft tissue and/or skeletal integrity |  |  |  |  |  |  |  |  |
| Medical diagnosis of patient |  |  |  |  |  |  |  |  |
| Imaging needed prior to spinal **mobilisations** |  |  |  |  |  |  |  |  |
| Informed consent from a guardian |  |  |  |  |  |  |  |  |
| Informed assent from the patient |  |  |  |  |  |  |  |  |
| Mechanism of injury |  |  |  |  |  |  |  |  |

|  |  | **Cervical** |  |  | **Thoracic** |  |  | **Lumbar** |
| --- | --- | --- | --- | --- | --- | --- | --- | --- |
|  | <2 | 2-12 | <18 | <2 | 2-12 | <18 | <2 | 2-12 <18 |
| Patient presentation (e.g., agitation, engagement, state of arousal) |  |  |  |  |  |  |  |  |
| Patient’s tolerance to handling |  |  |  |  |  |  |  |  |
| Posture exercises and self **mobilisations** are not enough to treat effectively |  |  |  |  |  |  |  |  |
| Therapist’s knowledge of techniques and when to use them appropriately |  |  |  |  |  |  |  |  |


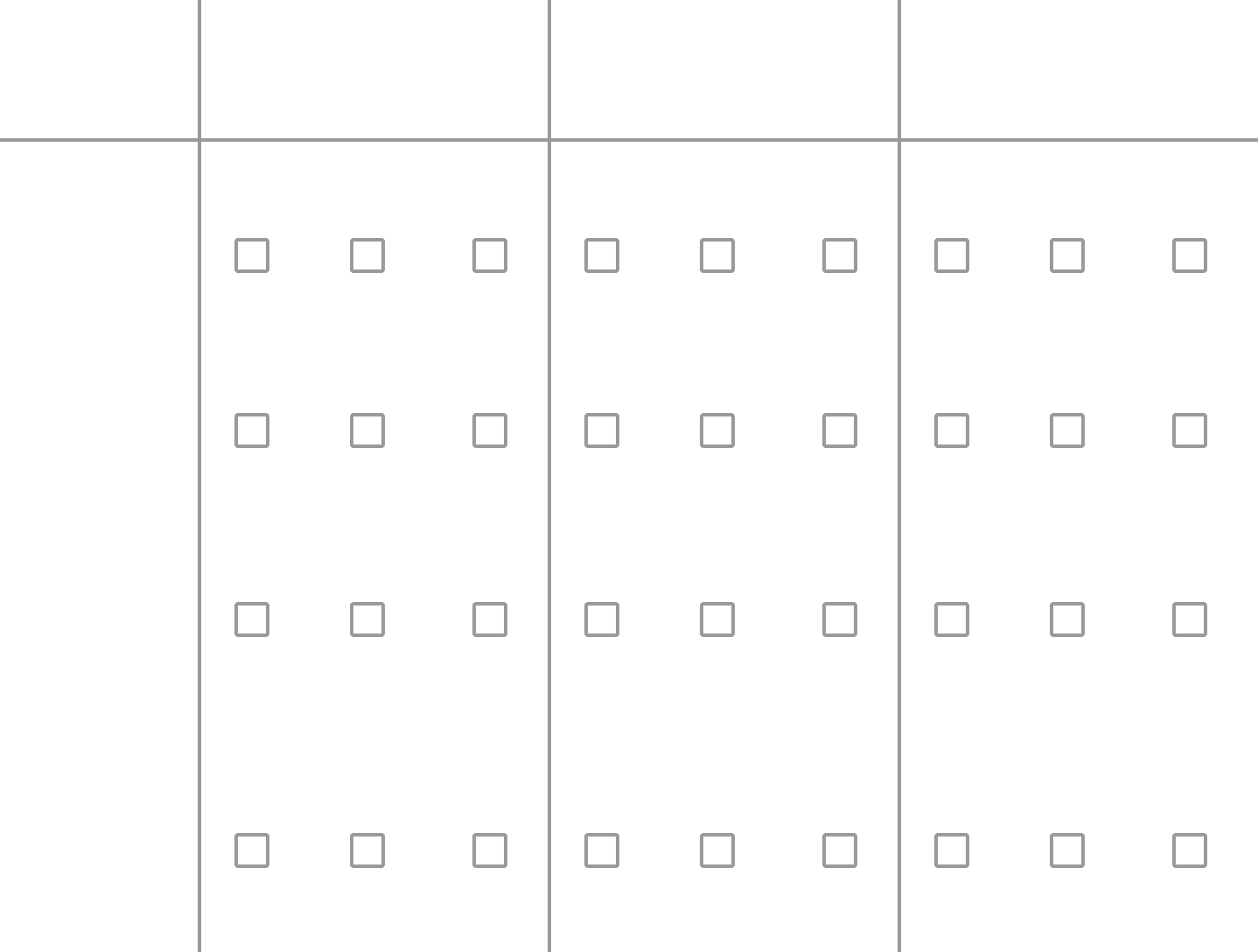
Please select the **FACTORS** that you believe would AFFECT a therapist’s decision to use or not use spinal **MANIPULATIONS** among the specific ages and spinal levels.


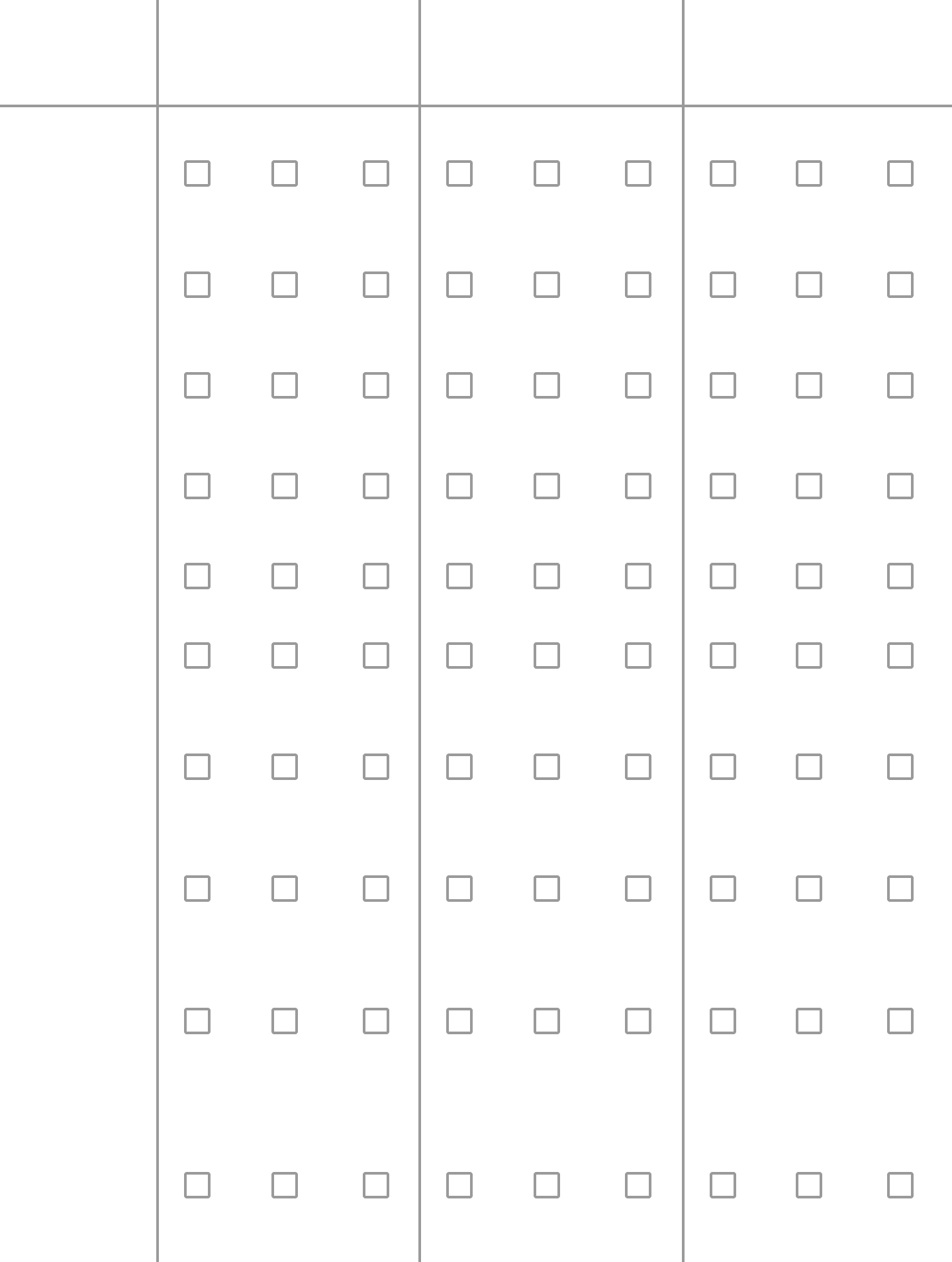


|  |  | **Cervical** |  |  | **Thoracic** |  |  | **Lumbar** |
| --- | --- | --- | --- | --- | --- | --- | --- | --- |
|  | <2 | 2-12 | <18 | <2 | 2-12 | <18 | <2 | 2-12 <18 |
| Concerns regarding soft tissue and/or skeletal integrity |  |  |  |  |  |  |  |  |
| Medical diagnosis of patient |  |  |  |  |  |  |  |  |
| Imaging needed prior to spinal **manipulations** |  |  |  |  |  |  |  |  |
| Informed consent from a guardian |  |  |  |  |  |  |  |  |
| Informed assent from the patient |  |  |  |  |  |  |  |  |
| Mechanism of injury |  |  |  |  |  |  |  |  |
| Patient presentation (e.g., agitation, engagement, state of arousal) |  |  |  |  |  |  |  |  |
| Patient’s tolerance to handling |  |  |  |  |  |  |  |  |
| Posture exercises and self **mobilisations** are not enough to treat effectively |  |  |  |  |  |  |  |  |
| Therapist’s knowledge of techniques and when to use them appropriately |  |  |  |  |  |  |  |  |

Please select the **BARRIERS** that you believe would PREVENT a therapist from using spinal **MOBILISATIONS** among the specific ages and spinal levels.


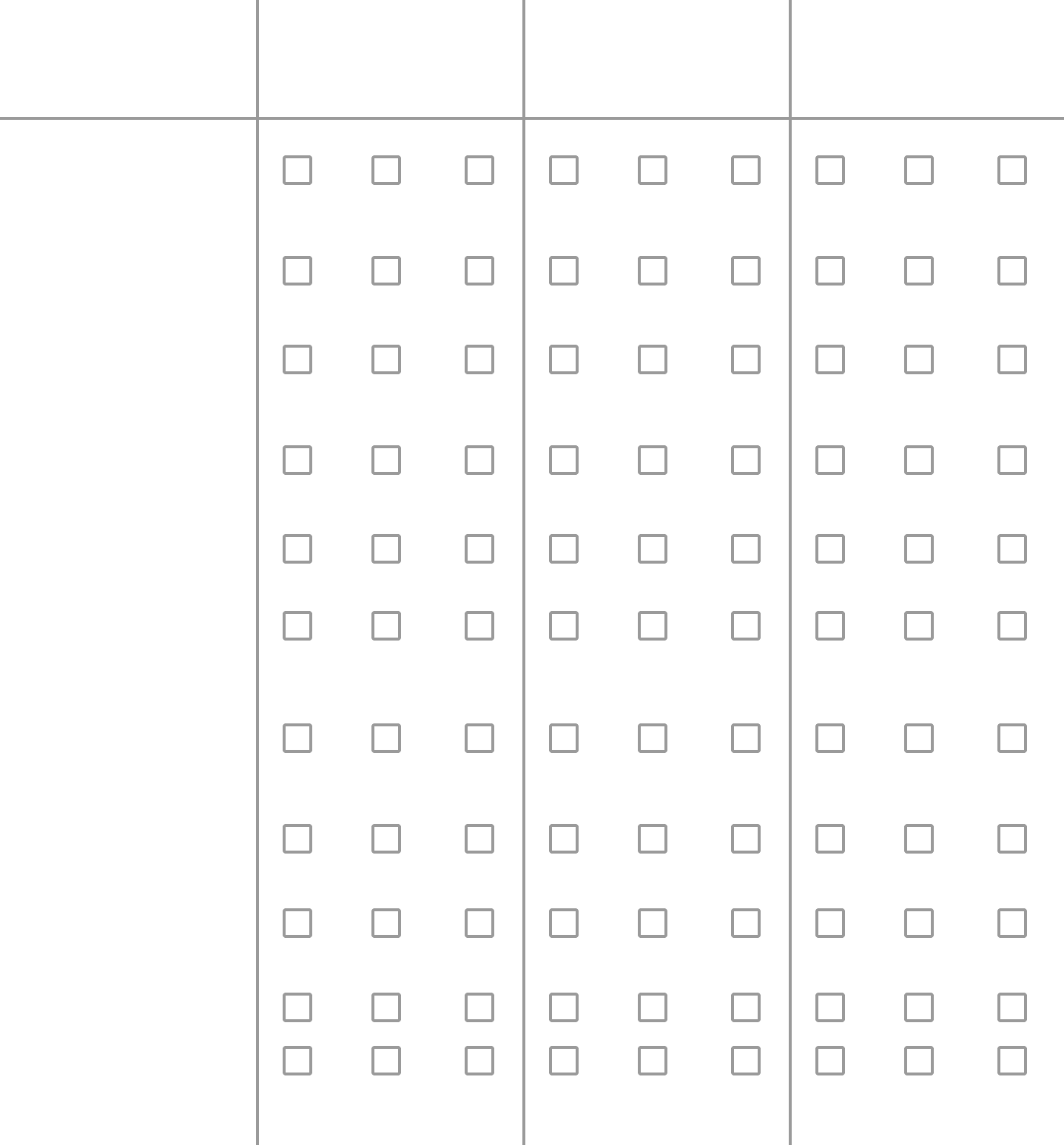


|  |  | **Cervical** |  |  | **Thoracic** |  |  | **Lumbar** |
| --- | --- | --- | --- | --- | --- | --- | --- | --- |
|  | <2 | 2-12 | <18 | <2 | 2-12 | <18 | <2 | 2-12 <18 |
| It is inappropriate to use **mobilisations** for this age |  | | | | | | | |
| There are no preventative barriers to **mobilisations** |  |  |  |  |  |  |  |  |
| Bias against using  **mobilisations** |  |  |  |  |  |  |  |  |
| Fear of injuring the patient (e.g., causing neurovascular or bony injury) |  |  |  |  |  |  |  |  |
| Fear of litigation |  |  |  |  |  |  |  |  |
| Lack of communication (among patient, guardian, and therapist) |  |  |  |  |  |  |  |  |
| Lack of evidence to support the use of **mobilisations** in the age group |  |  |  |  |  |  |  |  |
| Lack of guardian consent |  |  |  |  |  |  |  |  |
| Lack of patient |  |  |  |  |  |  |  |  |
| assent |  |  |  |  |  |  |  |  |
| Lack of experience in paediatric development |  |  |  |  |  |  |  |  |
| Lack of knowledge/training in techniques |  |  |  |  |  |  |  |  |

|  |  | **Cervical** |  |  | **Thoracic** |  |  | **Lumbar** |
| --- | --- | --- | --- | --- | --- | --- | --- | --- |
|  | <2 | 2-12 | <18 | <2 | 2-12 | <18 | <2 | 2-12 <18 |
| Lack of mentorship |  | | | | | | | |
| Medical diagnosis is not clearly defined |  |  |  |  |  |  |  |  |
| Poor understanding/cognition on the part of patient or guardian |  |  |  |  |  |  |  |  |
| The precision of examination to inform clinical reasoning |  |  |  |  |  |  |  |  |
| **Mobilisation** is not within the scope of practice |  |  |  |  |  |  |  |  |


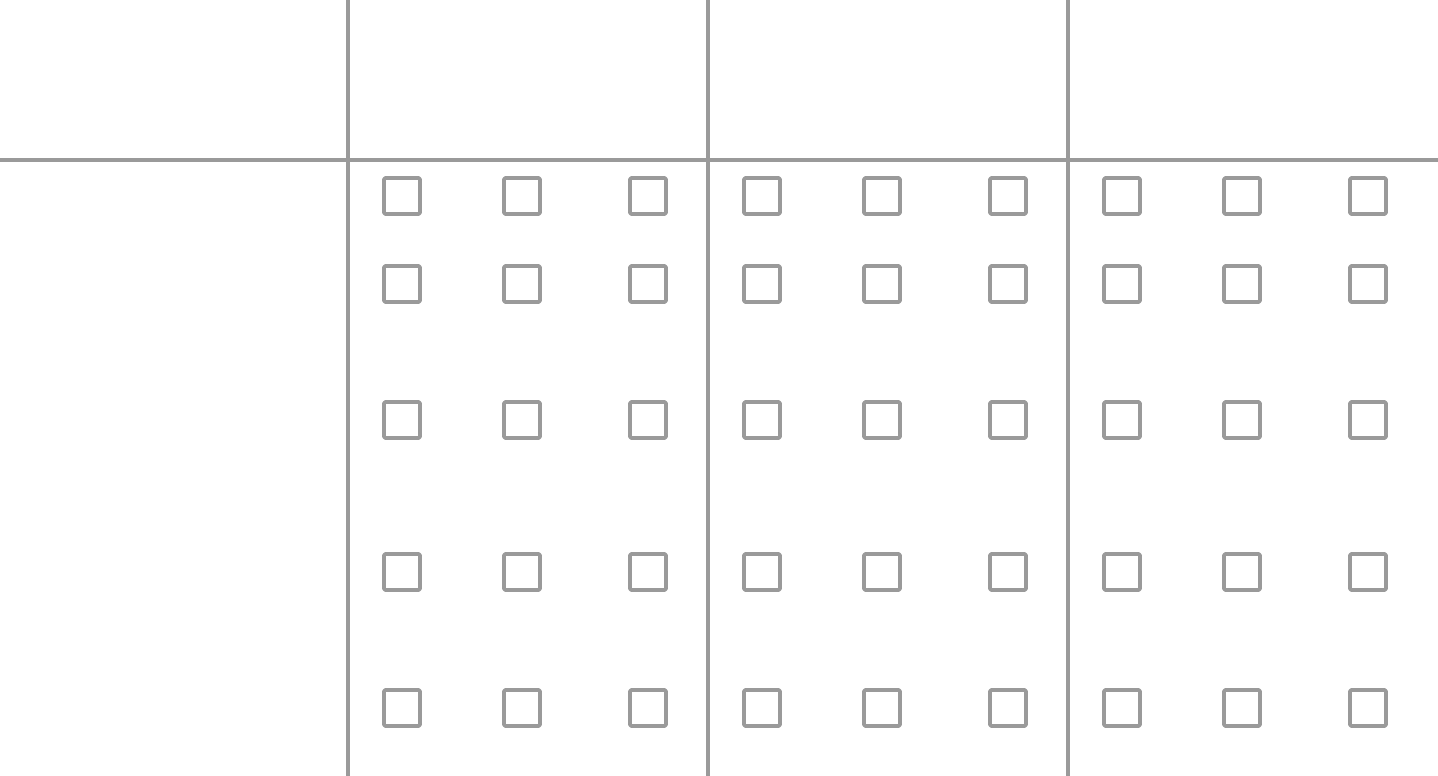
Please select the **BARRIERS** that you believe would PREVENT a therapist from using spinal **MANIPULATIONS** among the specific ages and spinal levels.


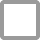

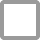

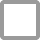

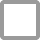

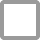

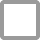

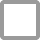

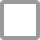

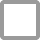

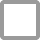

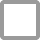

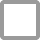

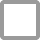

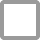

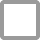

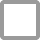

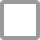

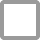

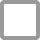

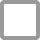

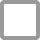

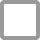

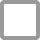

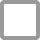

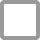

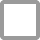

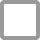


**Cervical**

**Thoracic**

**Lumbar**

<2

2-12

<18

<2

2-12

<18

<2

2-12

<18

It is inappropriate to use **manipulations** for this age

There are no preventative barriers to **manipulations**

Bias against using

**manipulations**

|  |  | **Cervical** |  |  | **Thoracic** |  |  | **Lumbar** |
| --- | --- | --- | --- | --- | --- | --- | --- | --- |
|  | <2 | 2-12 | <18 | <2 | 2-12 | <18 | <2 | 2-12 <18 |
| Fear of injuring the patient (e.g., causing neurovascular or bony injury) |  |  |  |  |  |  |  |  |
| Fear of litigation |  |  |  |  |  |  |  |  |
| Lack of communication (among patient, guardian, and therapist) |  |  |  |  |  |  |  |  |
| Lack of evidence to support the use of **manipulations** in the age group |  |  |  |  |  |  |  |  |
| Lack of guardian consent |  |  |  |  |  |  |  |  |
| Lack of patient |  |  |  |  |  |  |  |  |
| assent |  |  |  |  |  |  |  |  |
| Lack of experience in paediatric development |  |  |  |  |  |  |  |  |
| Lack of knowledge/training in techniques |  |  |  |  |  |  |  |  |
| Lack of mentorship |  |  |  |  |  |  |  |  |
| Medical diagnosis is not clearly defined |  |  |  |  |  |  |  |  |
| Poor understanding/cognition on the part of patient or guardian |  |  |  |  |  |  |  |  |
| The precision of examination to inform clinical reasoning |  |  |  |  |  |  |  |  |
| **Manipulation** is not within the scope of practice |  |  |  |  |  |  |  |  |


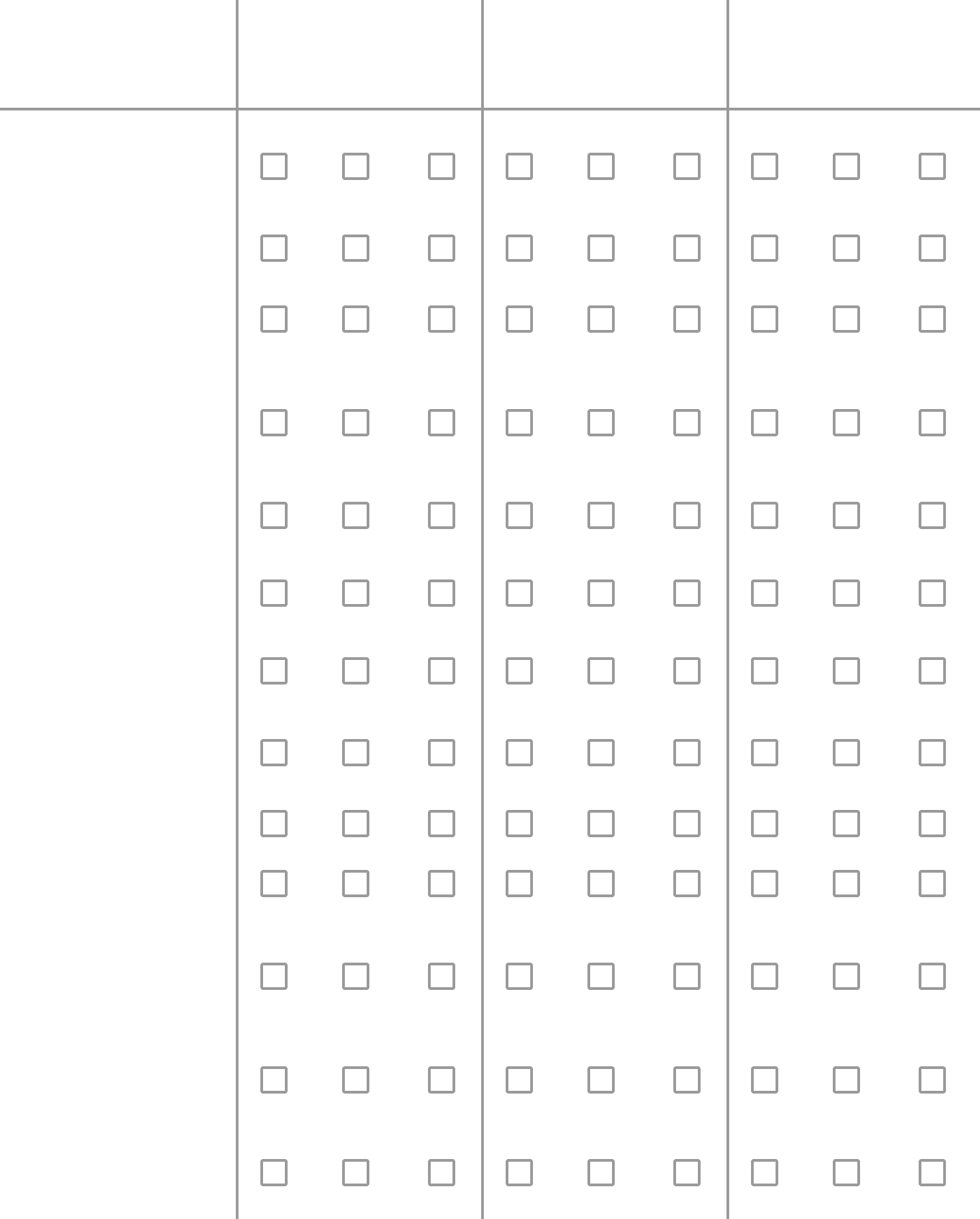


Do you wish to be included in the final round of this Delphi investigation?


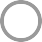


No


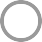


Yes

Powered by Qualtrics
