# Supplementary material for: Perceived factors and barriers affecting physiotherapists’ decision to use spinal manipulation and mobilisation among infants, children, and adolescents: an international survey
Source: J Man Manip Ther. 2024 Jun 28;32(3):295–303. doi: 10.1080/10669817.2024.2363033 (PMC11216267; doi:10.1080/10669817.2024.2363033)
Supplement: Appendix A Survey_Round_1 Clean.docx [file YJMT_A_2363033_SM3350.docx]

Survey Round 1

# Inclusion/Exclusion

Clicking the **“Yes”** button below indicates that you are 18 years of age or older, and indicates your consent to participate in this survey.


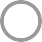
 Yes, I am 18 or older and I consent to participate to this survey


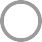
 No, I AM NOT 18 or older and I DO NOT consent to participate to this survey

Are you able to read/understand English?

Yes, I am able to read/understand English

No, I AM NOT able to read/understand English

Please select which category(ies) fit(s) best your practice as a physiotherapist? (Select **ALL** that apply)

Specialising in the exclusive treatment of paediatric orthopaedic conditions utilising manual / manipulative therapy as a part of your management strategy

With specialised education in musculoskeletal / manipulative therapy with at least 5 years of clinical experience

Specialising in paediatrics with exposure to the use of manual therapy techniques and with at least 5 years of clinical experience

With a research background, including at least some publications in the area of paediatrics and/or manipulative therapy

None

In the survey below:

Infants = birth to < 2 years

Children = 2 to 12 years

Adolescents = 13 to <18 years

What age range is your clinical expertise/knowledge focused on?

Patients > 18 years Patients birth to < 18 years Both

On average, what percentage of your weekly physiotherapy caseload consists of infants **(birth to < 2 years)**?

I do not typically treat infants (birth to < 2 years)

0-5%

6-10%

11-20%

21-50%

50-75%

76-100%

On average, what percentage of your weekly physiotherapy caseload consists of children **(2 to 12 years)**?

I do not typically treat children (2 to 12 years) 0-5% 6-10%

11-20%

21-50%

50-75%

76-100%

On average, what percentage of your weekly physiotherapy caseload consists of adolescents **(13 to < 18 years)**?

I do not typically treat adolescents (13 to < 18 years) 0-5%

6-10%

11-20%

21-50%

50-75%

76-100%

For the purposes of the following questions, **mobilisations** are *“continuum of skilled passive movements that are applied at varying speeds and amplitudes to joints, muscles or nerves with the intent to restore optimal motion, function, and/or to reduce pain”* (IFOMPT Standards Document, 2016)

Please indicate which **barriers** *prevent* physiotherapists’ decision to apply **spinal mobilisations** to infants, children and adolescents? (select **ALL** that apply)

Fear of injuring patient

Fear of litigation

Lack of knowledge or training on technique

Infants = birth to < 2 years


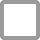

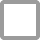

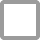


## Barriers – **Spinal Mobilisations**

Children = 2 to 12 years


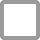

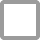

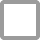


Adolescents = 13 to < 18 years


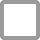

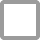

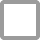


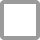

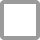

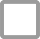


Lack of evidence to support use of spinal mobilisations


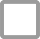

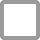

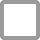
Lack of experience in pediatric development


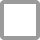

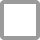

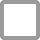
Bias against using spinal mobilisation


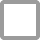

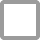

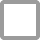
Spinal mobilisation is not within the scope of practice in this country


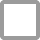

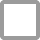

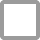
No preventive barriers

# If any, how many more OTHER BARRIERS do you wish to identify?

0

What OTHER **barriers** *prevent* physiotherapists from applying **spinal**

***mobilisations*** not mentioned above? (Up to 10 were offered to respondents)

Infants = birth to < 2 years

Children = 2 to 12 years

Adolescents = 13 to

< 18 years

Other barriers 1

Please indicate which **factors** *influence* physiotherapists’ decision to apply **spinal mobilisations** to infants, children and adolescents? (select **ALL** that apply)

## **Factors - Spinal Mobilisations**

Infants = birth to

< 2 years

Children = 2 to 12 years

Adolescents = 13 to < 18 years


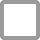

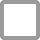

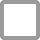
Determining elements affecting the decision to use or not use spinal mobilisations


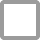

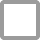

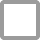
Patient presentation


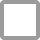

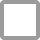

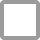
Tolerance to handling


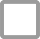

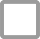

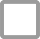
Diagnosis


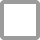

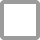

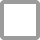
Mechanism of injury


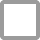

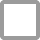

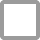
Therapist’s knowledge of spinal mobilisations and/or when to use technique


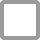

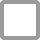

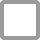
Imaging needed prior to performing spinal mobilisations


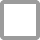

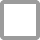

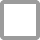
Concerns regarding soft tissue and/or skeletal integrity


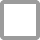

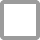

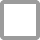
No inﬂuencing factors

# If any, how many more OTHER FACTORS do you wish to identify?

0

What OTHER **factors** *influence* physiotherapists’ decision to apply **spinal mobilisations** not mentioned above? (Up to 10 were offered to respondents)

Infants = birth to <2 years

Children = 2 to 12 years

Adolescents = 13 to

<18 years

Other factors 1

For the purposes of the following questions, **manipulation** is *“a passive, high velocity, low amplitude thrust applied to a joint complex within its anatomical limit with the intent to restore optimal motion, function, and/or to reduce pain”*

(IFOMPT Standards Document 2016)

Please indicate which **barriers** *prevent* physiotherapists from applying **spinal manipulations** to infants, children and adolescents? (select **ALL** that apply)

## **Barriers - Spinal Manipulations**

Infants = birth to

< 2 years

Children = 2 to 12 years

Adolescents = 13 to < 18 years


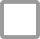

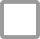

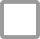

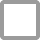

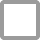

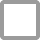
Fear of injuring patient Fear of litigation


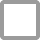

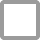

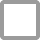
Lack of knowledge or training on technique


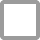

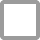

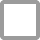
Lack of evidence to support use of spinal manipulations


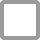

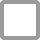

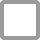
Lack of experience in pediatric development


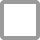

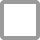

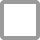
Bias against using spinal manipulations


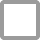

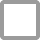

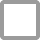
Spinal manipulations are not within the scope of practice in this country


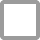

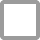

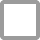
No preventive barriers

# If any, how many more OTHER BARRIERS do you wish to identify?

0

What OTHER **barriers** *prevent* physiotherapists from applying **spinal manipulations** not mentioned above? (Up to 10 were offered to respondents)

Infants = birth to < 2 years

Children = 2 to 12 years

Adolescents = 13 to

< 18 years

Other barriers 1

Please indicate which **factors** *influence* physiotherapists’ decision to apply **spinal manipulations** to infants, children and adolescents? (select **ALL** that apply)

## **Factors - Spinal manipulations**

Infants = birth to

< 2 years

Children = 2 to 12 years

Adolescents = 13 to < 18 years


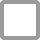

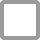

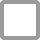
Determining elements affecting the decision to use or not use spinal manipulations


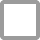

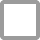

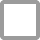
Patient presentation


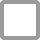

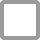

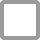
Tolerance to handling


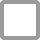

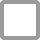

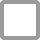
Diagnosis


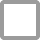

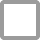

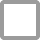
Mechanism of injury


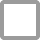

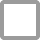

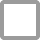
Therapist’s knowledge of spinal manipulations and/or when to use technique


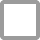

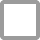

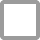
Imaging needed prior to performing spinal manipulations


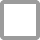

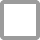
Concerns regarding soft tissue and/or skeletal integrity

No inﬂuencing factors

# If any, how many more OTHER FACTORS do you wish to identify?

0

What OTHER **factors** *influence* physiotherapists’ decision to apply **spinal manipulations** not mentioned above? (Up to 10 were offered to respondents)

Infants < 2 years

Other factors 1

Children = 2 to 12 years

Adolescents = 13 to

< 18 years

What is your age?

What is your gender?

Male

Female

Non-binary/third gender

Prefer not to say

What is your educational background in physiotherapy? (select **ALL** that apply)

Non-university physiotherapy diploma

- Bachelor of Science in physiotherapy / Bachelor of Physiotherapy

Master of Science in physiotherapy / Master of Physiotherapy

- Clinical Doctorate of physiotherapy / Masters (Extended) of Physiotherapy
- Academic Post-Professional Doctorate Degree (PhD, ScD, EdD, DHsc, etc)
- Advanced certiﬁcation in orthopedic manual therapy
- Advanced certification in paediatrics

Other

Please enter other education, certification and/or advanced proficiency in physiotherapy.

Please enter the number of years you have been as a practising physiotherapist.

Please enter the country where you are currently practising.

What setting do you primarily teach physical therapy in? (select **ALL** that apply)

I am currently not teaching

- Academic setting (university / physiotherapy school)

Continuing Education (post-professional)

- Residency/Fellowship
- Clinical setting (clinical instructor)

Other

Please enter other physiotherapy educational setting where you teach

Please consider submitting your e-mail address so we may contact you directly in order to further participate in the Delphi investigation. The completion of this Delphi investigation could provide consensus among international physiotherapists regarding the use of spinal mobilisations and manipulations for specific paediatric conditions and ages. Each Round of the Delphi investigation will be strictly voluntary, but equally vital to complete this research.

Your e-mail address will be used only for the purposes of this Delphi investigation.

If you have questions regarding this survey, please contact:

Jenifer Dice, PT, ScD [jenifer.dice@gmail.com](mailto:jenifer.dice@gmail.com)

Do you wish to be included in the future rounds of this Delphi investigation?

YES NO

Clicking "YES" connects you to another survey to enter your e-mail address.

Please contact Jenifer Dice (jenifer.dice@gmail.com) if the next page does not load.

Your responses are very valuable to this study. Thank you for taking the time to ﬁll out this survey.

If answering *NO* or *NONE* to initial screening questions:

From your responses, it has been determined that you do not qualify to participate in this survey. Thank you for your interest in this study.
